# Study on Biopharmaceutics Classification and Oral Bioavailability of a Novel Multikinase Inhibitor NCE for Cancer Therapy

**DOI:** 10.3390/ijms15057199

**Published:** 2014-04-25

**Authors:** Yang Yang, Chun-Mei Fan, Xuan He, Ke Ren, Jin-Kun Zhang, Ying-Ju He, Luo-Ting Yu, Ying-Lan Zhao, Chang-Yang Gong, Yu Zheng, Xiang-Rong Song, Jun Zeng

**Affiliations:** 1 State Key Laboratory of Biotherapy, West China Hospital, Sichuan University, Chengdu 610041, Sichuan, China; E-Mails: yyde2013@163.com (Y.Y.); hexuan9014@163.com (X.H.); luodyu@163.com (L.-T.Y.); zhaoyinglan@scu.edu.cn (Y.-L.Z.); chygong14@yahoo.com.cn (C.-Y.G.); zhengyu_iris@163.com (Y.Z.); 2 West China School of Pharmacy, Sichuan University, Chengdu 610041, Sichuan, China; E-Mails: fanchunmeiok@163.com (C.-M.F.); ymyzjk@163.com (J.-K.Z.); heyingju@tom.com (Y.-J.H.); 3 Department of Pharmaceutical Sciences, University of Nebraska Medical Center, Omaha, NE 68198, USA; E-Mail: renkemallee@gmail.com; 4 Department of Anesthesiology, West China Hospital, Sichuan University, Chengdu 610041, Sichuan, China

**Keywords:** biopharmaceutics classification system, intrinsic dissolution rate, permeability, bioavailability, multikinase inhibitor

## Abstract

Specific biopharmaceutics classification investigation and study on phamacokinetic profile of a novel drug candidate (2-methylcarbamoyl-4-{4-[3- (trifluoromethyl) benzamido] phenoxy} pyridinium 4-methylbenzenesulfonate monohydrate, NCE) were carried out. Equilibrium solubility and intrinsic dissolution rate (IDR) of NCE were estimated in different phosphate buffers. Effective intestinal permeability (*P*_eff_) of NCE was determined using single-pass intestinal perfusion technique in rat duodenum, jejunum and ileum at three concentrations. Theophylline (high permeability) and ranitidine (low permeability) were also applied to access the permeability of NCE as reference compounds. The bioavailability after intragastrical and intravenous administration was measured in beagle dogs. The solubility of NCE in tested phosphate buffers was quite low with the maximum solubility of 81.73 μg/mL at pH 1.0. The intrinsic dissolution ratio of NCE was 1 × 10^−4^ mg·min^−1^·cm^−2^. The *P*_eff_ value of NCE in all intestinal segments was more proximate to the high-permeability reference theophylline. Therefore, NCE was classified as class II drug according to Biopharmaceutics Classification System due to its low solubility and high intestinal permeability. In addition, concentration-dependent permeability was not observed in all the segments, indicating that there might be passive transportation for NCE. The absolute oral bioavailability of NCE in beagle dogs was 26.75%. Therefore, dissolution promotion will be crucial for oral formulation development and intravenous administration route will also be suggested for further NCE formulation development. All the data would provide a reference for biopharmaceutics classification research of other novel drug candidates.

## 1. Introduction

Targeted small-molecular inhibitors are promising antitumor candidates compared to the traditional chemotherapy drugs due to their high efficiency and low toxicity [1]. Their common formulations are oral preparations with good compliance. The essential parameters controlling oral drug absorption are the permeability of the drug through the gastrointestinal (GI) membrane and the drug solubility/dissolution in GI milieu [2,3]. Biopharmaceutics Classification System (BCS) is a modern tool to characterize drug permeability and solubility/dissolution [4,5]. According to BCS, drugs are characterized into four categories: Class I—high permeability, high solubility; Class II—high permeability, low solubility; Class III—low permeability, high solubility; Class IV—low permeability, low solubility. By using BCS system, some drug-drug interactions could be prevented and the *in vivo* bioavailability testing of the immediate release dosage forms of class I and III drugs could be bypassed [6]. Up till now, there is rare systemic report about specific classification method according to BCS, especially for a novel drug candidate.

According to the Food and Drug Administration (FDA) guidance for biowaiver request of immediate-release solid oral dosage forms, a drug substance is considered as high soluble when the highest dose strength is soluble in ≤250 mL of aqueous media over the pH range of 1.0–7.5 [7]. However, the choice of different dose has a great impact on the dose/solubility ratio and then affects the classification of drugs [8]. In addition, as a static equilibrium parameter, solubility cannot effectively describe the dynamic dissolution behavior especially in the physiological condition [9]. There are lots of experimental models which have been developed to determine the intestinal absorptive potential of a drug and its absorption mechanism [10], among which it is crucial that the achieved data have a high correlation with the absorption characteristics in human beings [11].

**Figure 1.**
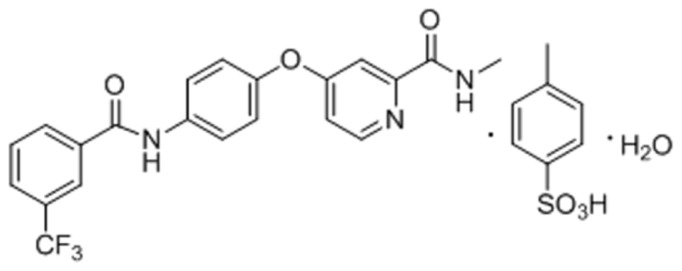
Chemical structure of NCE.

A novel multikinase inhibitor, 2-methylcarbamoyl-4-{4-[3-(trifluoromethyl) benzamido] phenoxy} pyridinium 4-methylbenzenesulfonate monohydrate (NCE) (shown in Figure 1) is selected as a model drug. NCE was designed and synthesized by our group. In the previous study, we had proved NCE was a promising drug candidate for cancer therapy. It can potently inhibit angiogenesis-related tyrosine kinase vascular endothelial growth factor receptor 2 (VEGFR2), fibroblast growth factor receptor 2 (FGFR2) and platelet-derived growth factor receptor (PDGFR) at rates of 97%, 65% and 55%, respectively, at a concentration of 10 μM in biochemical kinase assays [12]. In addition, NCE showed more selective inhibition of VEGF-stimulated human umbilical vein endothelial cells (HUVECs) proliferation *in vitro* experiments [12]. As a novel drug candidate, there is a need to characterize the solubility and intestinal permeability of NCE to further facilitate formulation development.

Measuring the rate and extent of drug absorbed into the blood circulation is a key step for developing novel drugs. Having a comprehensive knowledge of the drug bioavailability can facilitate guiding the development and production of pharmaceutical preparations. Moreover, bioavailability study can provide the basis for assessing the rationality of drug prescription design. Taking into account the above information, the purpose of the study was to investigate the biopharmaceutics classification of NCE according to BCS and evaluate its pharmacokinetic profile. These studies will aid in further formulation development of NCE and provide a reference for the research of other novel drug candidates.

## 2. Results and Discussion

### 2.1. Solubility and Intrinsic Dissolution Ratio of NCE

The equilibrated solubility of NCE in phosphate buffers was shown in Table 1. The aqueous solubility of NCE over the pH range of 1.0–7.4 was quite low with a maximum solubility of 81.73 μg/mL at pH 1.0. From its chemical structure (Figure 1), the parent structure of NCE is apparently a weak base and formulated as a methylbenzene sulfonate salt. The p*K*_a_ value of its conjugate acid has been determined as 2.86, which is attributed to the pyridine group (p*K*_a_ = 5.25 [13]). Thus, NCE would be ionized at low pH (pH = 1.0) with a high solubility and a mainly molecular state at high pH (such as 5.0, 6.8 and 7.4) with a low solubility. According to the Henderson-Hasselbach equation [14], NCE would maintain unionized within the pH range of GI tract. The partition coefficient (log*P* value) was calculated with 3.1 ± 0.04 in 0.1 M HCl solution (pH = 1.0) and 4.20 ± 0.07 in phosphate buffer solution (PBS) 6.8 solution, indicating that NCE is highly lipid soluble and could be well absorbed *in vivo*.

Intrinsic dissolution ratio is defined as the dissolution rate of a pure drug substance under the condition of constant surface area, agitation or stirring speed, pH and ionic strength of the dissolution medium. As a rate characterization, IDR data has a good correlation with *in vivo* drug dissolution rate [15]. Because of the low solubility of NCE in the tested phosphate buffers, sodium dodecyl sulfate (SDS) was added to increase the solubility to meet the sink condition which refers that the concentration of drug in release medium is far smaller than its saturation concentration. Three different SDS concentrations were tested. The solubilities of NCE were 54.46 (0.4% SDS), 131.83 (1% SDS) and 255.91 (2% SDS) μg/mL. The volume of the release medium in this experiment was 900 mL, thus the introduction of 1% SDS into the medium was enough to achieve sink condition.

**Figure 2.**
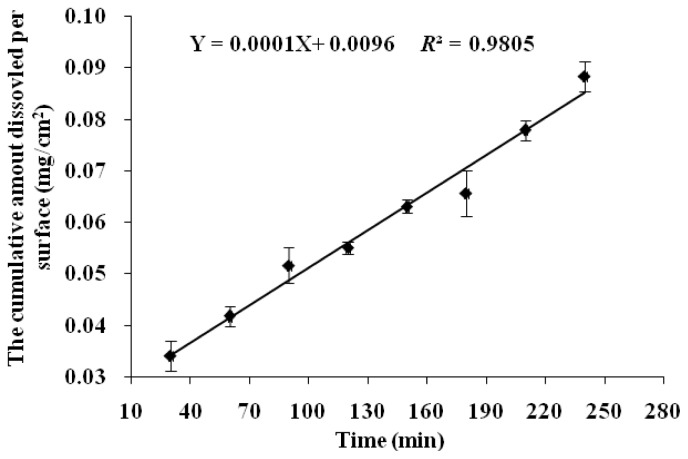
Intrinsic dissolution rate (IDR) of NCE in 0.1 mol/mL phosphate buffer (pH = 6.8).

The IDR was estimated by the dissolution data from the initial 4 h because it got close to the saturate concentration after 4 h with a decreased dissolution rate (Figure 2). The IDR of NCE was calculated to be 1 × 10^−4^ mg·min^−1^·cm^−2^, which was below the borderline value of 1 mg·min^−1^·cm^−2^ [15,16]. Therefore, NCE was classified as a compound with low solubility.

### 2.2. Stability of NCE in Perfusion Buffers

The concentration variation rates of NCE for all the samples in stability study were less than 5%, (as shown in Table 2) indicating that the drug was stable in perfusion buffers at these time points. Thus, determination of the samples should be performed within 6 h at room temperature or 48 h at 4 °C.

### 2.3. The Effect of Intestinal Site and NCE Concentration on Intestinal Permeability

*In vitro* and *in vivo* methods are frequently used to predict the drug absorption in small intestine [17]. Furthermore, *in vivo* method was significantly superior to *in vitro* method because there was no damange to the lymphatic and circulatory systems of the studied tissues. Among the *in vivo* methods, there are *in vivo* circulation methods and single pass intestine perfusion (SPIP) methods. As for *in vivo* circulation method, the long-term experiment process and high perfusion speed can cause damage to the intestinal mucosa, leading to an increase in drug absorption. Thus, the measured value and the true value have a large deviation. SPIP approach is the most frequently used method that provides *in situ* experimental conditions simulating oral administration [16,18–20]. By possessing a preserved microenvironment around the intestinal membrane, SPIP method is able to accurately characterize the drug absorption [21] in different small intestine regions. In addition, the data have a high correlation with the absorption characteristics in human beings [11]. The effective permeability (*P*_eff_) values (effective permeability) of human and rats had been proved to be highly relevant [16,22]. Therefore, the permeability investigation in rat intestine was necessary, which could be used to predict the oral absorption of NCE in human.

Several methods can be applied to calculate effective permeability of NCE in rat intestine. Such as the gravimetric method, the phenol red or ^14^C-PEG marked ways to indicate the change of perfusion volume [23]. However, the phenol red itself can be absorbed partly by the intestine. Also, it can affect the intestinal transport and analysis of certain compounds. The ^14^C-PEG marker exits radioactivity and security issues. Thus, gravimetric method was chosen to be applied in this study.

As previously described, due to the poor solubility of NCE in the perfusion buffer, different concentrations of SDS (0.4%, 1% and 2%, *w*/*v*) were added into the perfusion buffer to improve the solubility of NCE. The concentration of NCE in perfusion buffer containing 0.4% SDS was high enough to conduct the following intestinal permeability study. Additionally, according to preliminary experiments, SDS with concentrations higher than 1% would be toxic to rats [24]. Thus, 0.4% SDS was finally chosen to be added into the final perfusion buffer solution to improve the solubility of NCE.

In order to investigate whether 0.4% SDS has any effect on intestinal absorption of NCE, 2 μg/mL of NCE with and without 0.4% SDS was applied to three intestinal segments using the rat perfusion model in this study. The effective permeability of NCE in duodenum, jejunum and ileum was shown in Table 3. The *P*_eff_ values of NCE with and without SDS in any intestinal segment were similar (*p* > 0.05). No significant enhancement or reduction absorption was found in any intestinal segments, indicating that 0.4% SDS had no effects on intestinal absorption of NCE.

To achieve a more comprehensive evaluation of drug permeability, a high permeability compound (theophylline) and a low permeability compound (ranitidine) were applied in this study [8,25]. The absorption mechanism of these two compounds was passive diffusion [16,26,27], suggesting that the drug concentration had no effects on its absorption. Thus, 40 μg/mL of theophylline, ranitidine and NCE perfusion solution were prepared for the SPIP study.

The effective permeability (*P*_eff_) of NCE, theophylline and ranitidine in duodenum, jejunum and ileum was shown in Tables 3 and 4. As shown in Table 4, significant difference was found among these three drugs in the same intestinal segment (*p* < 0.05). The *P*_eff_ value of NCE was higher than ranitidine and lower than theophylline in any intestinal segment. As described previously, theophylline was a high permeability drug and ranitidine was a low permeability drug [25]. The *P*_eff_ values of NCE were more proximate to that of theophylline with high permeability, suggesting that NCE has a high permeability. The permeability coefficients of NCE obtained from SPIP study in three intestinal segments at three different concentrations, with the minimum of *P*_eff_ (0.20 × 10^−4^ cm/s) found in jejunum and the maximal *P*_eff_ (0.56 × 10^−4^ cm/s) found in duodenum, were comparable to propranolol (*P*_eff_ of propranolol: 0.30–0.75 × 10^−4^ cm/s) which was a widely used reference compound for high permeability evaluation [4,28]. Hence, NCE was considered to be well absorbed in the whole intestine and categorized as a high permeability drug. In conclusion, NCE was categorized as BCS class II drug due to its low solubility and high permeability. All the data would provide a reference for biopharmaceutics classification research of other novel drug candidates.

The effective permeability (*P*_eff_)of different concentrations of NCE in duodenum, jejunum and ileum was shown in Table 5. The *P*_eff_ values of NCE in different intestinal segment at the same concentration were similar (*p* > 0.05). No significant intestinal site dependent absorption was found at all concentrations, indicating that NCE was absorbed in the whole intestine without any site specific absorption sites. In addition, when the concentration of NCE increased from 2–40 μg/mL, the *P*_eff_ values in the same intestinal segment had no significant difference (*p* > 0.05). Generally, when the drug’s permeability does not change with the change of its concentrations, its transportation mechanism was passive diffusion according to Thomas J Cook [29]. Hence, the transportation mechanism of NCE in intestine was passive diffusion.

### 2.4. Pharmacokinetic Parameters of NCE in Beagle Dogs

**Figure 3.**
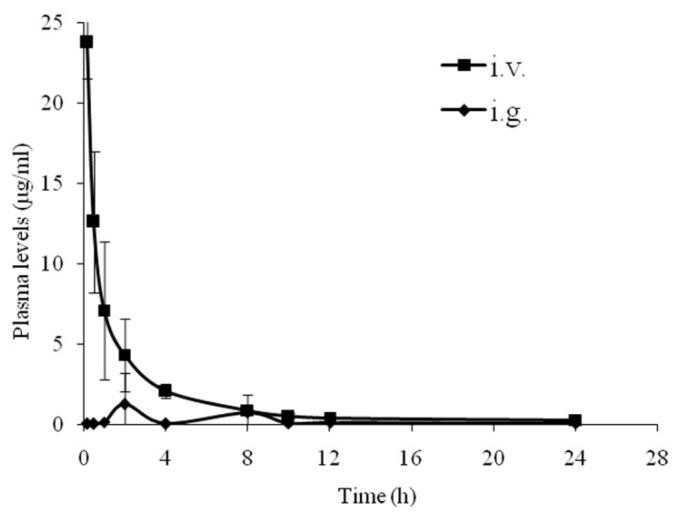
Plasma concentration (ng/mL) *versus* time (h) profiles of NCE after i.v. and i.g. administration of 30 mg/kg in beagle dogs (*n* = 3).

The mean plasma concentrations *versus* time profiles of NCE following intragastrical (i.g.) or intravenous (i.v.) administration in beagle dogs were shown in Figure 3. The pharmacokinetic parameters were presented in Table 6. As shown in Figure 3, the plasma concentration of NCE dropped rapidly after i.v. administration. It was almost below the detection limit after 12 h. After i.g. administration, there were two plasma NCE concentration peak observed (2 and 8 h). This phenomenon might be attributed to the enterohepatic circulation, two absorption sites at gastrointestinal tract or dosage form factors of NCE [30]. The i.v./i.g. *C*_max_ ratio was about 11.10, whereas the AUC_0−_*_t_* *ratio* was 4.19. Moreover, the elimination phase of the i.g. curve almost overlapped that of the i.v. curve. The absolute bioavailability of NCE after oral administration was 26.75% ± 13.77%. Our previous investigation displayed that the absolute oral bioavailability of NCE was 44.18% ± 16.87% in rats [31]. It can be concluded that the pharmacokinetic profiles between dogs and rats had no significant difference due to the similar absolute oral bioavailability (*p* = 0.11). However, the multiple peaks phenomenon in beagle dogs was not observed in rats, suggesting the species differences in NCE tolerability which was consistent with the published literatures [32].

## 3. Experimental Section

### 3.1. Materials

NCE (purity > 98%, by high performance liquid chromatography) was provided by State Key Laboratory of Biotherapy (Chengdu, China). Theophylline was purchased from Shanghai Yingyuan Chemical Co., Ltd. (Shanghai, China). Ranitidine and Hanks’ Balanced Salts solution (HBSS) were purchased from Sigma-Aldrich (St. Louis, MO, USA). HEPES was purchased from Amresco Co., LLC. (Solon, OH, USA). HPLC grade water was obtained from a Milli-Q water purification system (Millipore Co., Ltd., Molsheim, France). Other chemicals were of HPLC or analytical grade.

### 3.2. Solubility Determination

Different phosphate buffers with a pH range of 1.0–7.4 were prepared as follows. Momobasic potassium phosphate (KH_2_PO_4_, 0.2 M) was prepared by dissolving 27.22 g of KH_2_PO_4_ in water and diluting with water to 1000 mL. Sodium hydroxide (NaOH, 0.2 M) was prepared by dissolving 11.8 g of NaOH in water and diluting with water to 1000 mL. Then, we placed 50 mL of the momobasic potassium phosphate solution in a 200 mL volumetric flask, added the specified volume of the sodium hydroxide solution and added water to volume.

The equilibrium solubilities of NCE were determined in 0.1 mol/mL phosphate buffers with a pH range of 1.0–7.4. An excess amount of NCE was put into the buffer, placed in a 37 °C water bath and shaken for 3 days at a rate of 100 rpm. Samples were centrifuged at a rate of 1.3 × 10^4^ rpm for 10 min and then measured by HPLC (Waters e2695 Separations Module, Milford, MA, USA). Each experiment was performed in triplicates.

The partition coefficient (log*P* value) is related closely to drug’s absorption *in vivo*. Furthermore, it can help to get a better understanding of drug’s physicochemical properties. The 1-octanol/water partition coefficient of NCE was measured by a shake-flask method. Both the 1-octanol and aqueous buffer solution were saturated before performing the experiments. Solutions of about 3.3 mM NCE were prepared in aqueous 0.1 M HCl (pH = 1.0) solution and PBS buffer (pH = 6.8) solutions. Then, 1 mL of 1-octanol was added to 9 mL of the aqueous NCE solution in glass flasks. The mixtures were then stirred in a mechanical shaker for 6 h (37 °C). Samples were left in water baths and kept at the appropriate temperature (37 °C) for at least 72 h. After that, the concentrations in the aqueous phase and 1-octanol phase were determined by HPLC, respectively. The partition coefficients were calculated by the following equation. All the partitioning experiments were performed in at least triplicate.

(1) $$P_{1-\text{octanol}/\text{water}}=\text{lg }(C_{1-\text{octanol}}/C_{\text{water}})$$

where *P*_1-octanol/_*_water_* is the partition coefficient value; *C*_1-octanol_ is the drug’s concentration in 1-octanol phase; *C*_water_ is the drug’s concentration in aqueous solution.

### 3.3. Dissolution-Based Classification Intrinsic Dissolution Ratio (IDR) Measurement

A modified IDR measurement method was carried out [33]. In brief, 50 mg NCE was compressed under an average compression force of 3.0 MPa for 1 min to make a non-disintegrating compact using a 5 mm die and punch. The surface area of the compact was 0.1963 cm^2^. Compacts were placed in a molten beeswax-mold in a way that only one side could come in contact with the dissolution medium. Dissolution study was conducted employing USP II dissolution apparatus using 900 mL phosphate buffer (0.1 mol/mL, pH = 6.8) with 1% SDS (*w*/*v*) at 37 ± 1 °C. The paddle rotating speed was 75 rpm. Samples were collected every 30 min for a period of 8 h and analyzed by HPLC. Samples were tested in triplicates.

### 3.4. Stability Study

Storage conditions were investigated to guarantee the samples were stable before HPLC analysis. The stability of NCE was evaluated in the perfusion solution (pH 6.5). Four aliquots of the NCE solutions were stored at room temperature for 0, 2, 4 or 6 h, respectively. Another three were stored at 4 °C for 0, 24 or 48 h, respectively. All the samples were centrifuged at a rate of 1.3 × 10^4^ rpm for 10 min and then measured by HPLC. The sample concentrations were compared.

### 3.5. In Situ Single-Pass Perfusion (SPIP) Experiments

The blank perfusion buffer solution was composed of 9.80 g Hanks’ Balanced Salts, 5.96 g 4-2(Hydroxyethyl) piperazine-1-ethanesulfonic acid (HEPES), 1.16 g NaCl, 3.50 g d-glucose and 0.37 g NaHCO_3_. The pH was adjusted to 6.5. Due to the poor solubility of NCE in the perfusion buffer, SDS was added to increase its solubility. The perfusate containing 2 μg/mL of NCE with and without 0.4% SDS was applied to three intestinal segments to investigate the effect of SDS on intestinal absorption of NCE. To examine whether there was any concentration-dependent characteristics or site specific absorption of NCE, 2, 20 and 40 μg/mL of NCE containing 0.4% SDS (*w*/*v*) were also studied in three intestinal segments using the same rat perfusion model. In addition, 40 μg/mL theophylline (high permeability) and ranitidine (low permeability) [8] were applied to assess the permeability level of NCE.

Male Sprague-Dawley rats (170–230 g) were obtained from the Laboratory Animal Center, Sichuan University (Chengdu, China). Prior to the experiment, animals were fasted for at least 18 h with free access to water. All animal studies were approved and supervised by the State Key Laboratory of Biotherapy Animal Care and Use Committee (Sichuan University, Chengdu, Sichuan, China). Then, single pass intestine perfusion studies were conducted according to the literature [34]. In brief, rats were anaesthetized by pentobarbital sodium (i.p., 300 mg/kg) and placed on a homeothermic pad to maintain their body temperature. After making a 3–4 cm midline abdominal incision, the small intestinal was exposed. The duodenum, jejunum and ileum were isolated as follows: duodenum segment beginning from 1 cm away from pylorus, jejunum segment beginning from 20 cm away from pylorus and ileum segment beginning at the site 20 cm upwards caecum. Each intestinal segment was approximately 10 cm long. After removing the intestinal content by rinsing with warm saline, two glass cannulas were inserted to the distal and proximal end of the segment. Then, the intestinal segments of interest were connected to a perfusion pump and the outlet tubing was placed at the same height as the inlet. The intestine was handled carefully to maintain an intact blood supply.

The segments were rinsed with perfusate at the rate of 1.0 mL/min for 10 min, and then perfused at the rate of 0.2 mL/min for 30 min to maintain a steady-state. The time was set zero immediately when the perfusion started. The outlet perfused samples were collected every 15 min until 120 min. Then, the samples were centrifuged at 1.3 × 10^4^ rpm for 10 min. The supernatant was analyzed by HPLC. The length of tested segments was measured and rats were euthanized after experiment.

### 3.6. Pharmacokinetic Studies in Beagle Dogs

Six healthy beagle dogs (7.97 ± 0.73 kg) were randomly assigned into two groups. NCE was given to the dogs by intragastrical (i.g.) and intravenous (i.v.) administration at a dosage of 30 mg/kg. Blood was collected from the forelimb vein of the dogs at 0.25, 0.5, 0.75, 1, 2, 3, 4, 6, 8, 10, 12 and 24 h. Plasma samples were obtained by centrifuging blood at 3000 rpm for 10 min and were stored at −20 °C. Before analysis, acetonitrile was added into the plasma and the mixture was vortexed for 5 min to precipitate the protein. Then, the samples were centrifuged for 10 min at a speed of 1.3 × 10^4^ rpm. The supernatant was collected and the solvent was removed by pressure blowing concentrator. Then, mobile phase was added to dissolve the residual. The concentrations of NCE in plasma were determined by HPLC.

### 3.7. HPLC Analytical Method

The concentrations of NCE, theophylline and ranitidine in all samples were determined using Waters e2695 HPLC system with an Ultimate C_18_ column (250 mm × 4.6 mm, 5 μm). The mobile phases of NCE, theophylline and ranitidine were acetonitrile-0.1% (*v*/*v*) methanoic acid water solution (65/35, *v*/*v*), water-acetonitrile (90/10, *v*/*v*), and phosphate buffer (pH = 3.5)-methanol (72/28, *v*/*v*), respectively. All the flow rates for three drugs determination were set at 1.0 mL/min and column temperatures were 30 °C. The detection wavelengths for the three drugs were set at 270, 271 and 314 nm, respectively.

### 3.8. IDR Calculation

To evaluate the IDR of NCE, the cumulative amount dissolved per surface unit of the compact was plotted against time. The slope of the linear region (*R*^2^ ≥ 0.95) was taken as the intrinsic dissolution rate. IDR was calculated by the following equation as described previously [34].

(2) $$\text{IDR}=\left(\frac{dw}{dt}\right)\left(\frac{1}{S}\right)=\frac{DCs}{h}$$

where IDR is the intrinsic dissolution rate (mg/min/cm^2^); *dw* is the change in drug dissolved (mg); *dt* is the change in time (min); *S* is the surface area of the compact (cm^2^); *D* is diffusion coefficient (cm^2^/s); *Cs* is the solubility (mg/cm^3^) and *h* is the stagnant layer thickness (cm).

### 3.9. Effective Permeability Coefficient (P_eff_) Calculation

*P*_eff_ is the quantitative parameter estimating the passage rate of a solute across a membrane. It was calculated from the steady-state concentration of NCE in the perfusate [35]. The drug concentration in the perfusate was corrected for changes in the water flux. Meanwhile, the density corrected gravimetric method was employed to calculate the net water flux across the incubated intestinal segment. A known volume of perfusate was weighed using an electronic weighing balance to determine the density of the collected samples. After correcting for water flux, the drug concentration in the effluent perfusate (*C_out(cor)_*), was calculated as following [36].

(3) $$C_{out(cor)}=C_{out}\times \frac{Q_{out}}{Q_{in}}$$

where *C_out_* is the concentration of tested drug in the effluent perfusates, *Q_in_* and *Q_out_* are the inlet and outlet flow rate (mL/min), respectively, which are adjusted for liquid density (mL/min).

The calculation of permeability across rat intestinal segment was performed from the intestinal perfusate samples collected over 15–120 min (steady state) using the following equation).

(4) $$P_{eff}=\frac{-Q_{\text{in}}\;\text{ln}(C_{out(cor)}/C_{in})}{2\pi rl}$$

where *P_eff_* is the effective permeability coefficient, *C_in_* is the input perfusate drug concentration, *r* is the effective lumen radius (cm), and *l* is the length of intestinal segment, 2*πrl* is the area of the mass transfer surface (cm^2^) within the intestinal segment which is assumed to be a cylinder area.

### 3.10. Pharmacokinetic Parameters Calculation

The pharmacokinetic parameters were calculated using the pharmacokinetics-intelligent analysis module of DAS 2.1.1 software. Total areas under the concentration-time curve (AUC) after i.g. and i.v. administration were determined by extrapolation from time 0−*t* (AUC_0−_*_t_*), respectively. The oral bioavailability of NCE was estimated using the ratio of AUC values following i.g. and i.v. administration.

### 3.11. Statistical Analysis

Data were analyzed by independent-samples *t*-test of statistical analysis software SPSS 16.0. Permeability coefficient results were expressed as mean ± standard deviation (SD). A *p* value of less than 0.05 was considered as statistical significance.

## 4. Conclusions

Based on our data, NCE was categorized as BCS class II drug due to its low solubility and high permeability. NCE was absorbed in the whole small intestine without a specific absorption site. There might be a passive transportation mechanism of NCE in the small intestine. The absolute bioavailability after i.g. administration in beagle dogs was 26.75% ± 13.77%. Thus, the improvement of NCE dissolution would be a key point for oral formulation development. To develop novel intravenous preparations with improved NCE water-solubility might be an alternative strategy, which might be a challenge because of its poor water-solubility.

## Figures and Tables

**Table 1.** Solubility of NCE in phosphate buffers (*n* = 3, mean).

Buffers pH	Solubility (μg/mL)	SD
1.0	81.73	0.13
5.0	08.50	0.04
6.8	09.30	0.06
7.4	10.00	0.09

**Table 2.** Stability of NCE in perfusion buffers.

Temperature	20 °C				4 °C	
		
	0 h	2 h	4 h	6 h	24 h	48 h
Detected (μg/mL)	19.83	19.66	19.49	19.32	19.16	18.99
Rate of change (%)	-	0.86	1.70	2.55	3.38	4.21

**Table 3.** The effect of 0.4% sodium dodecyl sulfate on intestinal absorption of NCE using single-pass intestinal perfusion method. The data were presented as mean ± SD (*n* = 3 for each group).

Perfusate (μg/mL)	*P*_eff_ (10^−4^ cm/s)		

	Duodeum	Jejunum	Ileum
NCE	0.26 ± 0.29	0.25 ± 0.24 ^N.S.^	0.23 ± 0.18 ^N.S.^
0.4% SDS + NCE	0.26 ± 0.25 ^N.S.^	0.25 ± 0.22 ^N.S.^	0.22 ± 0.15 ^N.S.^

N.S., not significantly different to the group of duodenum segment of NCE or 0.4% SDS + NCE at the same concentration or not significantly different to the group of NCE in the same intestinal segment.

**Table 4.** Effective permeability of NCE, theophylline and ranitidine using single-pass intestinal perfusion method. The data were presented as mean ± SD (*n* = 6 for each group).

Perfusate (40 μg/mL)	*P*_eff_ (10^−4^ cm/s)		

	Duodeum	Jejunum	Ileum
NCE	0.49 ± 0.29 *	0.56 ± 0.26 ^N.S.^ *	0.52 ± 0.28 ^N.S.^ *
Theophylline	0.74 ± 0.02 *	0.64 ± 0.01 ^N.S.^ *	0.63 ± 0.04 ^N.S.^ *
Ranitidine	0.15 ± 0.07 *	0.19 ± 0.07 ^N.S.^ *	0.17 ± 0.02 ^N.S^ *

N.S., not significantly different to the group of duodenum segment of NCE, theophylline or ranitidine at the same concentration;

* *p*<0.05, compared to the other two groups in the same intestinal segment.

**Table 5.** Effective permeability of NCE using single-pass intestinal perfusion method. The data were presented as mean ± SD (*n* = 6 for each group).

Perfusate (μg/mL)	*P*_eff_ (10^−4^ cm/s)		

	Duodeum	Jejunum	Ileum
2	0.21 ± 0.18	0.20 ± 0.30 ^N.S.^	0.23 ± 0.25 ^N.S.^
20	0.34 ± 0.12	0.30 ± 0.15 ^N.S.^	0.29 ± 0.10 ^N.S.^
40	0.49 ± 0.29	0.56 ± 0.26 ^N.S.^	0.52 ± 0.28 ^N.S.^

N.S., not significantly different to the group of duodenum segment of NCE at the same concentration or not significantly different to the group of 2 μg/mL of NCE in the same intestinal segment.

**Table 6.** Pharmacokinetic parameters of NCE in beagle dogs. *C*_max_, peak plasma concentration; *T*_max_, time of peak; AUC_0−_*_t_, area under the plasma concentration-time curve from 0–t* h; MRT, mean residence time; F, absolute bioavailability.

Route	Beagles	*C*_max_ (μg/L)	*T*_max_ (h)	*T*_1/2_ (h)	AUC_0−_*_t_* (mg/L·h)	MRT (h)	F (%)
i.v.	Beagle 1	26.01	0.23	6.39	56.25	1.84	-
	Beagle 2	23.67	0.30	3.56	34.41	1.68	-
	Beagle3	21.56	0.22	9.23	25.11	1.35	-
	mean ± SD	23.75 ± 2.23	0.25 ± 0.04	6.39 ± 2.83	38.59 ± 15.99	1.62 ± 0.25	-

i.g.	Beagle 4	12.19	2.00	12.28	6.86	10.79	12.19
	Beagle 5	0.86	1.73	17.34	13.61	10.57	39.56
	Beagle 6	2.06	2.00	14.92	7.16	11.19	28.51
	mean ± SD	2.14 ±1.32	1.91 ± 0.15	14.85 ± 2.53	9.21 ± 3.82	10.85 ± 0.31	26.75 ± 13.77
